# Pan-cancer analysis of *ALK* mutation and its association with tumor immunogenicity and the efficacy of immune checkpoint blockade

**DOI:** 10.1016/j.gendis.2025.101701

**Published:** 2025-05-28

**Authors:** Zhiyang Huang, Jiajun Chen, Yan Huang, Hong Zhao, Bin Zhao

**Affiliations:** a Quanzhou First Hospital Affiliated to Fujian Medical University, Quanzhou, Fujian 362000, China; b The Cancer Center of the Fifth Affiliated Hospital of Sun Yat-Sen University, Zhuhai, Guangdong 519000, China

**Keywords:** *ALK*, Biomarker, Cancer, Immune checkpoint blockade, Tumor immunogenicity

## Abstract

Anaplastic lymphoma kinase (*ALK*) plays important roles in tumorigenesis and is involved in tumor immunogenicity through various pathways. Here, we conducted a comprehensive bioinformatic and clinical analysis on the characteristics of pan-cancer *ALK* mutation and its association with tumor immunity and the efficacy of immune checkpoint blockade. In 2930 patients with 11 tumor types treated with immune checkpoint inhibitors, the mutation of *ALK* indicated favorable overall survival (hazard ratio = 0.69; 95% confidence interval, 0.57–0.83; *p* < 0.001). We further developed and validated a nomogram to estimate the 12-month and 24-month survival probabilities after the initiation of immunotherapy. Moreover, multi-omics analysis on both intrinsic and extrinsic immune landscapes revealed that the mutation of *ALK* could enrich infiltration of immune cells, enhance tumor immunogenicity, and improve immune responses. In conclusion, *ALK* mutation is associated with promoted cancer immunity and can be treated as a biomarker for favorable outcomes in pan-cancer immune checkpoint blockade. These results have implications for treatment decision-making and developing immunotherapy for personalized care.

## Introduction

Applying immune checkpoint blockade in clinical practice has revolutionized cancer treatment since 2011.^1^ Immune checkpoint inhibitors targeting programmed cell death ligand 1 (PD-L1), programmed cell death protein 1 (PD-1), and cytotoxic T-lymphocyte-associated antigen 4 (CTLA-4) can significantly improve the overall survival and emerge as standard therapeutic approaches for various tumor types.^2^ However, although several biomarkers, including PD-L1 expression, tumor mutation burden, and microsatellite instability-high/deficient DNA mismatch repair, have been approved by the US FDA, it is still difficult to determine which patients should be offered immune checkpoint inhibitors currently.^3^^,^^4^

Anaplastic lymphoma kinase (ALK), a highly conserved receptor tyrosine kinase (RTK) in the insulin receptor superfamily, is essential for the development and growth of the nervous system.^5^ Oncogenic activation of *ALK* usually occurs in two different ways, namely somatic mutation and chromosomal rearrangement. These gain-of-function alterations play important roles in tumorigenesis through different signaling pathways.^5^ Although *ALK* overexpression has been identified in various cancer types, its relationship with tumor drivers is still unclear.^6^ Currently, it is well-established that the rearrangement of *ALK* has become a key element during clinical decision-making since tyrosine kinase inhibitors demonstrate robust anti-tumor activities.^7^ Indeed, five inhibitors, namely crizotinib (first-generation), ceritinib, alectinib, brigatinib (second-generation), and lorlatinib (third-generation), are approved by the FDA and have been widely used in treating *ALK-*rearranged tumors.^8^ However, numerous pieces of evidence reveal that there are significant heterogeneities in tumors harboring *ALK* rearrangement and *ALK* mutation.^5^^,^^6^^,^^8^ Hence, patients with *ALK*-mutant tumors often show resistance to tyrosine kinase inhibitors.^8^ For example, *ALK p. L1196M* and *G1269A* mutations can lead to the first-generation ALK-tyrosine kinase inhibitor resistance, while mutations like *ALK p. G1202R* account for half of the second-generation tyrosine kinase inhibitor resistance.^9^ Currently, the optimal therapeutic management strategies in patients with *ALK*-mutant tumors have not yet been fully investigated.

Most oncogenic drivers can profoundly impact the systemic and local immune landscape.^7^ It is reported that ALK is identified as a lymphoma-associated tumor antigen and affects CD8^+^ T cell responses.^10^ ALK can also regulate the release of various cytokines and their receptors, including interleukin (IL)-2, IL-10, interferon-gamma (IFN-γ), tumor necrosis factor alpha (TNF-α), and cluster of differentiation 30 (CD30),^6^ and the expression of immune checkpoints such as PD-L1.^11^ Hence, we speculate that the mutation of *ALK* results in the enrichment of tumor-associated neo-antigens and enhanced tumor immunity. The unique characteristics of *ALK* mutation make it a potential biomarker for cancer immunotherapy. Here, with accumulating publicly available data, we conducted a comprehensive bioinformatic and clinical analysis to retrospectively examine the association between *ALK* mutation and the outcomes in pan-cancer immunotherapy. Furthermore, a series of immunologic and genomic analyses were carried out to reveal the potential mechanisms underlying *ALK* mutant tumors.

## Material and methods

### Data collection

Patients with *ALK-*mutant tumor(s) who were treated with immune checkpoint inhibitors were extracted from 9 studies^12^, ^13^, ^14^, ^15^, ^16^, ^17^, ^18^, ^19^, ^20^ (Table 1). Among them, melanoma was investigated in 7 studies,^12^, ^13^, ^14^, ^15^, ^16^, ^17^^,^^20^ lung cancer in 4 studies,^12^^,^^15^^,^^18^^,^^19^ and multiple tumors in 2 studies.^12^^,^^15^ Whole-exome sequencing was applied for sequencing in 8 cohorts.^13^, ^14^, ^15^, ^16^, ^17^, ^18^, ^19^, ^20^ The MSK-IMPACT panel was employed in one study with multiple tumors.^12^ Agents targeting CTLA-4 were applied in 5 studies,^12^^,^^15^^,^^17^^,^^19^^,^^20^ and inhibitors targeting PD-1/PD-L1 were administered in 7 studies.^12^, ^13^, ^14^, ^15^, ^16^^,^^18^^,^^19^ Information regarding overall survival was collected directly from the original reports.

**Table 1:** Baseline characteristics of the eligible trials included in the immunotherapy analysis.

Authors (year)	Treatment agents	Cancer type	Detection method	*ALK* mutation	Number of patients
Discovery cohort					
Hugo et al (2016)^13^	Pembrolizumab/nivolumab	Melanoma	WES	Positive	7
				Negative	31
Liu et al (2019)^14^	Pembrolizumab/nivolumab	Melanoma	WES	Positive	22
				Negative	122
Miao et al (2018)^15^	Inhibitors targeting CTLA-4, PD-1, and PD-L1	Multiple tumors	WES	Positive	23
				Negative	226
Riaz et al (2017)^16^	Nivolumab	Melanoma	WES	Positive	5
				Negative	63
Van Allen et al (2015)^17^	Ipilimumab	Melanoma	WES	Positive	10
				Negative	100
Gandara et al (2018)^18^	Atezolizumab	Lung cancer	WES	Positive	10
				Negative	417
Ravi et al (2023)^19^	Inhibitors targeting CTLA-4 and PD-1 or in combination with chemotherapy	Lung cancer	WES	Positive	11
				Negative	298
Snyder et al (2014)^20^	Ipilimumab/tremelimumab	Melanoma	WES	Positive	5
				Negative	59
Validation cohort					
Samstein et al (2019)^12^	Inhibitors targeting CTLA-4, PD-1, and PD-L1	Multiple tumors	MSK-IMPACT panel	Positive	61
				Negative	1549

Note: WES, whole-exome sequencing; PD-L1, programmed cell death ligand 1; PD-1, programmed cell death protein 1; CTLA-4, cytotoxic T-lymphocyte-associated antigen 4.

Information regarding DNA methylation and sequencing, RNA expression, and clinicopathological characteristics of The Cancer Genome Atlas (TCGA) cohort was downloaded from https://gdc.cancer.gov/about-data/publications/pancanatlas. Key features used in this study including silent mutation rate, non-silent mutation rate, single nucleotide variant neoantigen, insertion/deletion (indel) neoantigen, lymphocyte fraction, leukocyte fraction, CD8^+^ T cell abundance, tumor-infiltrating lymphocyte regional fraction, T cell receptor (TCR) richness, B cell receptor (BCR) richness, TCR Shannon index, and BCR Shannon index, were calculated as previously described.^21^ The expression levels of related genes in Figure 4D–F were scaled separately according to mutation status, and the median values were derived to calculate the expression difference. Statistical analysis for comparisons between two groups was conducted using the Wilcoxon test.

**Figure 1:**
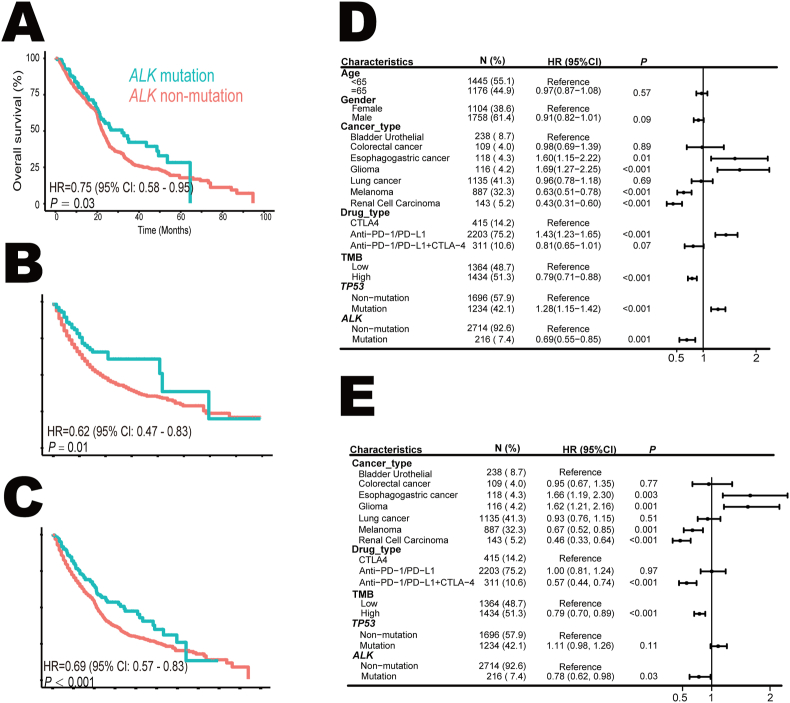
ALK mutation and overall survival (OS) in pan-cancer immune checkpoint blockade. **(A)** The Kaplan–Meier survival analysis stratified by *ALK* mutation status in 1406 cancer patients with 6 tumor types treated with immune checkpoint inhibitors in the discovery cohort. **(B)** The association between *ALK* mutation and OS in 1524 patients with 9 tumor types in the validation cohort. **(C)** Comparison of OS between patients with *ALK* mutation and patients with *ALK* non-mutation in 2930 subjects with 11 types of tumors treated with immune checkpoint inhibitors. **(D, E)** Univariate (D) and multivariate (E) Cox analysis of the association between *ALK* mutation and OS. ALK, anaplastic lymphoma kinase; CI, confidence interval; CTLA-4, cytotoxic T-lymphocyte-associated antigen 4; HR, hazard ratio; OS, overall survival; PD-1, programmed cell death protein 1; PD-L1, programmed cell death ligand 1; TMB, tumor mutation burden.

**Figure 2:**
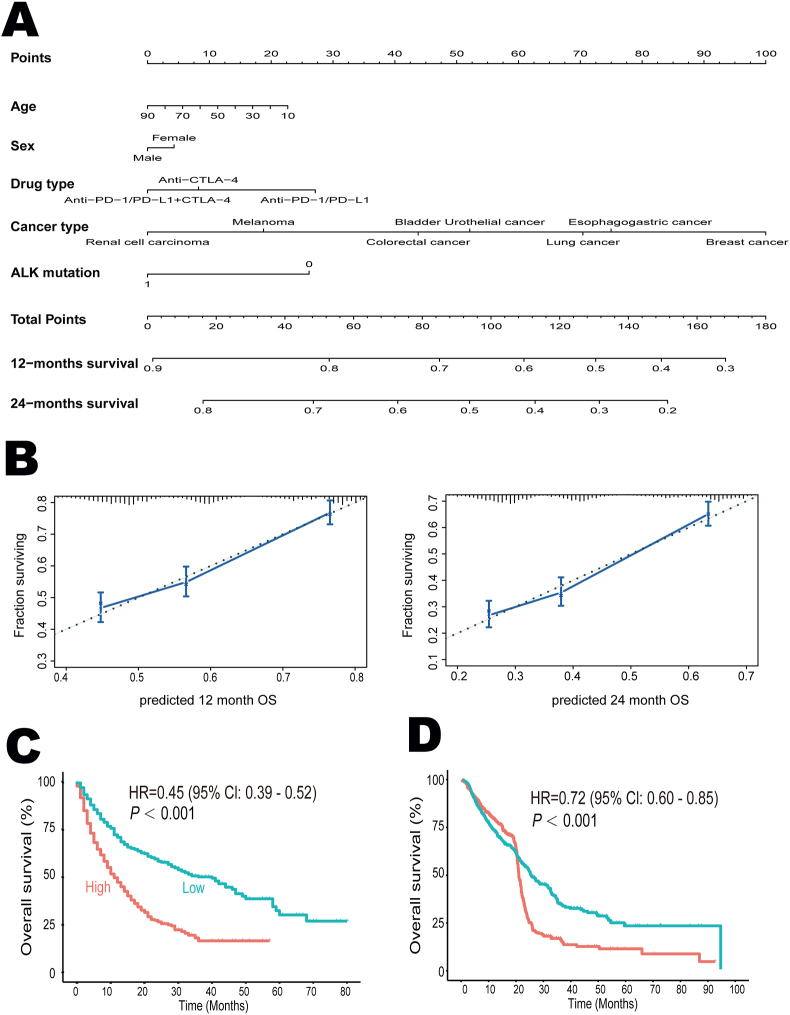
Survival predicting models after the initiation of immunotherapy. **(A)** The nomogram for predicting the 12- and 24-month survival. It can calculate overall survival from the date of immunotherapy start. To use the nomogram, we locate the “age” axis and draw a line up to the “point” axis to get a score associated with age, and repeat for the other features to get their scores. Then, we sum all scores and locate the final score on the “total point” axis, and draw a line to the “12-month survival” axis to get the 12-month overall survival probability. **(B)** The calibration plots for validation of the 12- and 24-month survival from the nomogram. The average predicted probability (*X* axis) was plotted against the observed Kaplan–Meier estimate in the subgroup (*Y* axis, 95 % CIs of the estimates are presented as vertical lines). The continuous line is the reference line, indicating what an optimal nomogram would be. **(C, D)** Based on the optimal cutoff value derived from the nomogram, a low score was associated with favorable overall survival in both the discovery cohort (C) and validation cohort (D). ALK, anaplastic lymphoma kinase; CI, confidence interval.

**Figure 3:**
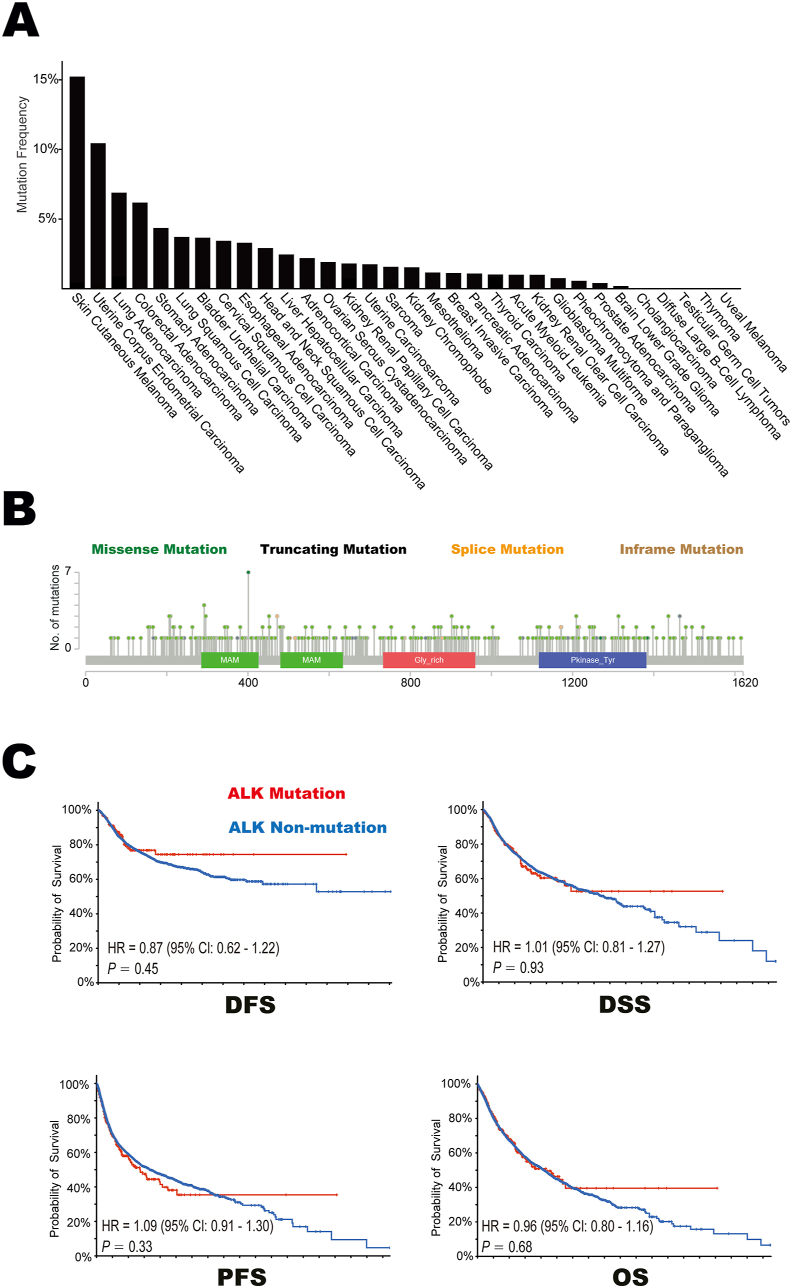
The characteristics of *ALK* mutation in 33 tumor types based on the TCGA cohort. **(A)** The mutation frequencies of *ALK* gene across 33 tumor types. **(B)** The subtypes and distributions of *ALK* somatic mutations. *X*-axis, amino acid; *Y*-axis, number of *ALK* mutations. MAM, MAM domain (285–426; 480–634); Gly_rich, glycine rich protein (733–960); Pkinase_Tyr, protein tyrosine kinase (1117–1382); green, missense mutation; black, truncating mutation; orange, spice mutation; brown, in-frame mutation. **(C)** Comparison of DFS, DSS, PFS, and OS between patients with *ALK* mutation and patients with *ALK* non-mutation in 10,953 subjects with 33 tumor types. ALK, anaplastic lymphoma kinase; DFS, disease-free survival; DSS, disease-specific survival; PFS, progression-free survival; OS, overall survival.

**Figure 4:**
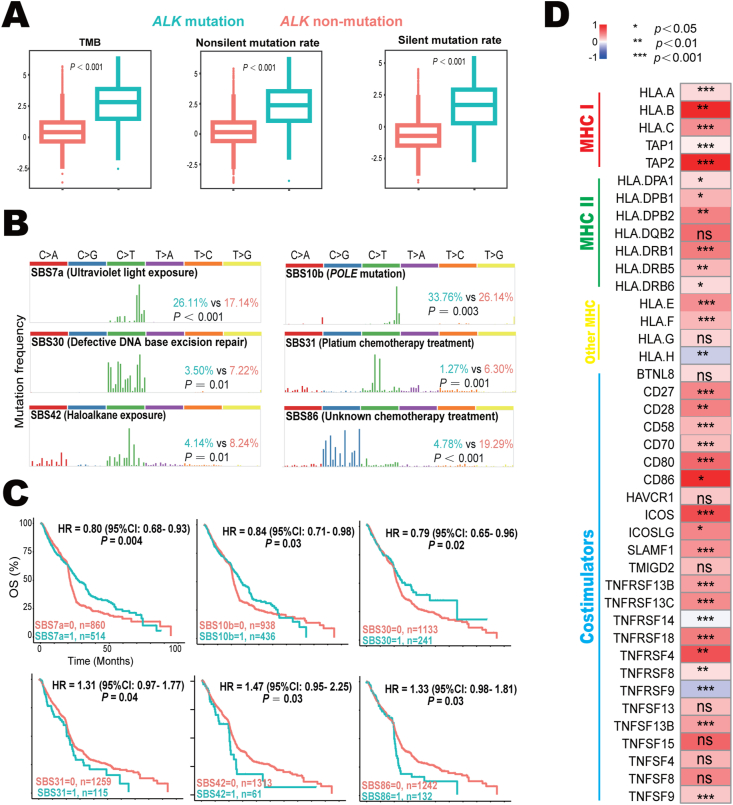
Intrinsic immunity of *ALK*-mutant and *ALK*-non-mutant tumors. **(A)** Comparison of TMB, non-silent mutation rate, and silent mutation rate between *ALK-*mutant and *ALK*-non-mutant tumors. **(B)** The illustrations of six identified SBS signatures related to *ALK* mutation and their frequencies in *ALK*-mutant and *ALK*-non-mutant tumors. Bold black, SBS signature and its known etiologies; green, frequency in *ALK*-mutant cancer; orange, frequency in *ALK*-non-mutant cancer. **(C)** The associations between identified mutation signatures and overall survival in pan-cancer immunotherapy. **(D)** Differences of 16 MHC-related antigen-presenting molecules (including 5 MHC I molecules, 7 MHC II molecules, and 4 other MHC molecules) and 25 co-stimulators between *ALK-*mutant and *ALK-*non-mutant tumors. The color scheme indicates the median value difference of the normalized gene expression between *ALK*-mutant and *ALK*-non-mutant tumors. ALK, anaplastic lymphoma kinase; MHC, major histocompatibility complex; SBS, single base substitution; TMB, tumor mutation burden.

### Assessment of immune signatures

The enrichment levels of 29 classical immune signatures were evaluated according to the single-sample gene set enrichment analysis (ssGSEA) method using the “GSVA” R package.^22^

### Deciphering mutational signatures

The “deconstructSigs” R package was applied to perform non-negative matrix factorization analysis of mutations and patterns of carcinoma evolution.^23^ The extracted mutation pattern was compared against the Catalog of Somatic Mutations in Cancer (COSMIC) based on cosine similarity.

### Generation and validation of the nomogram

Nomograms are widely used to predict outcomes quantitatively in oncology using critical predictive features. A calibration curve can be used to evaluate the similarity between the predictive and actual survival probability. The “rms” R package was used to generate both nomogram and calibration curves.

### Statistics

Survival curves were generated by the Kaplan–Meier method, and the log-rank test was used to evaluate the significance of differences. Hazard ratio (HR) and its 95 % confidence interval (CI) were calculated by the Cox proportional hazards model. The Kruskal–Wallis test, Wilcoxon test, or Chi-square test was used to analyze the associations among various categorical variables, depending on the context. All data processing and analysis were carried out by R software (version 4.2.1) and MedCalc (version 18.2.1). Two-sided *p*-values <0.05 were considered statistically significant.

## Results

### *ALK* mutation and overall survival in pan-cancer immune checkpoint blockade

To examine the impact of *ALK* mutation on pan-cancer immunotherapy, 1406 patients with 6 tumor types from 8 datasets were applied as the discovery cohort (Table 1). These patients were diagnosed as anal cancer (*n* = 1), bladder urothelial cancer (*n* = 27), head and neck cancer (*n* = 12), lung cancer (*n* = 791), melanoma (*n* = 574), and sarcoma (*n* = 1). *ALK*-mutant tumors were identified in 125 patients (8.9%) and were associated with longer overall survival (HR = 0.75; 95% CI: 0.58–0.95; *p* = 0.03; Fig. 1A). The validation cohort enrolled 1524 patients with 9 tumor types, including breast cancer (*n* = 41), bladder urothelial cancer (*n* = 211), colorectal cancer (*n* = 109), esophagogastric cancer (*n* = 118), glioma (*n* = 116), head and neck cancer (*n* = 129), lung cancer (*n* = 344), melanoma (*n* = 313), and renal cell carcinoma (*n* = 143). 91 patients (6.0%) with *ALK*-mutant tumors achieved favorable outcomes (HR = 0.62; 95% CI: 0.47–0.83; *p* = 0.01; Fig. 1B). Totally, in 2930 patients with 11 tumor types treated with immune checkpoint inhibitors, *ALK* mutation (*n* = 216; 7.4%) decreased the risk of death by 31% (HR = 0.69; 95% CI: 0.57–0.83; *p* < 0.001; Fig. 1C). Furthermore, both univariate (Fig. 1D) and multivariate (Fig. 1E) Cox proportional hazards analyses demonstrated that *ALK* mutation was an independent favorable predictor (HR = 0.79; 95% CI: 0.63–0.98; *p* = 0.03) after adjusting multiple confounding variables including age, sex, cancer type, drug type, tumor mutation burden, and *TP53* mutation status.

Next, we constructed a nomogram to estimate 12-month and 24-month survival based on the discovery cohort (Fig. 2A). Further examination of the calibrations for these predictions revealed that the performance of this cure-model-based nomogram was good (Fig. 2B), with the potential to estimate the survival probabilities after the initiation of immunotherapy. Moreover, we applied X-tile software to identify the optimal cutoff value (total points = 120) and categorized patients into low- and high-score subgroups. Low score was associated with better overall survival in both the discovery cohort (HR = 0.45; 95% CI: 0.39–0.52; *p* < 0.001; Fig. 2C) and the validation cohort (HR = 0.72; 95% CI: 0.60–0.85; *p* < 0.001; Fig. 2D).

### Characteristics of *ALK*-mutant tumors in the TCGA cohort

To investigate the underlying mechanisms between *ALK* mutation and immunotherapy, multi-omics information extracted from the TCGA cohort was explored. Overall, 323 of 10,953 enrolled patients (2.9%) harbored *ALK* somatic mutations. They were discovered in most tumor types (Fig. 3A), but the frequencies differed significantly among various tumors. Totally, 385 ALK mutations were identified; 328 (85.2%) were missense mutations, 36 (9.4%) were truncating mutations, 19 (4.9%) were splice mutations, and 2 (0.5%) were in-frame mutations. These mutations occurred in a dispersed manner throughout the whole sequence (Fig. 3B). Additionally, the outcomes were independent of *ALK* mutation in terms of disease-free survival (HR = 0.87; 95% CI: 0.62–1.22; *p* = 0.45), disease-specific survival (HR = 1.01; 95% CI: 0.81–1.27; *p* = 0.93), progression-free survival (HR = 1.09; 95% CI: 0.91–1.30; *p* = 0.33), and overall survival (HR = 0.96; 95% CI: 0.80–1.16; *p* = 0.68) (Fig. 3C). Giving all patients enrolled in TCGA cohort were treated with conventional management strategies, *ALK* mutation was not a universal prognostic biomarker for clinical outcomes in cancer but only a predictor for immune checkpoint blockade.

### Intrinsic immunity associated with *ALK*-mutant tumors

The key intrinsic immunity is usually referred to as high tumor immunogenicity, activation of the antigen-processing machinery, and overexpression of costimulatory molecules.^24^ As shown in Figure 4A, the mutation loads, including tumor mutation burden, non-silent mutation rate, and silent mutation rate, were significantly higher in *ALK*-mutant tumors. Next, we investigated if any specific mutant patterns were associated with the efficacy of immune checkpoint blockade in *ALK*-mutant patients. The frequencies of COSMIC reference signatures SBS7a (known etiology, ultraviolet light exposure; 26.1% *vs*. 17.1%; *p* < 0.001), SBS10b (*POLE* mutation; 33.8% *vs*. 26.1%; *p* = 0.003), SBS30 (defective DNA base excision repair; 3.5% *vs*. 7.2%; *p* = 0.01), SBS31 (platium chemotherapy treatment; 1.3% *vs*. 6.3%; *p* = 0.001), SBS42 (haloalkane exposure; 4.1% *vs*. 8.2%; *p* = 0.01), and SBS86 (unknown chemotherapy treatment; 4.8% *vs*. 19.3%; *p* < 0.001) were significantly different between *ALK*-mutant and *ALK*-non-mutant tumors (Fig. 4B). Further examination suggested that these signatures were robust biomarkers for overall survival in pan-cancer immune checkpoint blockade (Fig. 4C). Specifically, the presence of SBS7a (HR = 0.80; 95% CI: 0.68–0.93; *p* = 0.004), SBS10b (HR = 0.84; 95% CI: 0.71–0.98; *p* = 0.03), and SBS30 (HR = 0.79; 95% CI: 0.65–0.96; *p* = 0.02) indicted favorable overall survival. In contrast, SBS31 (HR = 1.31; 95% CI: 0.97–1.77; *p* = 0.04), SBS42 (HR = 1.47; 95% CI: 0.95–2.25; *p* = 0.03), and SBS86 (HR = 1.33; 95% CI: 1.00–2.99; *p* = 0.01) were negative predictors. The dysfunctions of major histocompatibility complex (MHC) were the main cause of tumor immune escape.^3^ *ALK* mutation was associated with increased expression of most MHC-related antigen-presenting molecules and co-stimulators (Fig. 4D).

### Underlying extrinsic immune mechanisms in *ALK*-mutant tumors

**Figure 5:**
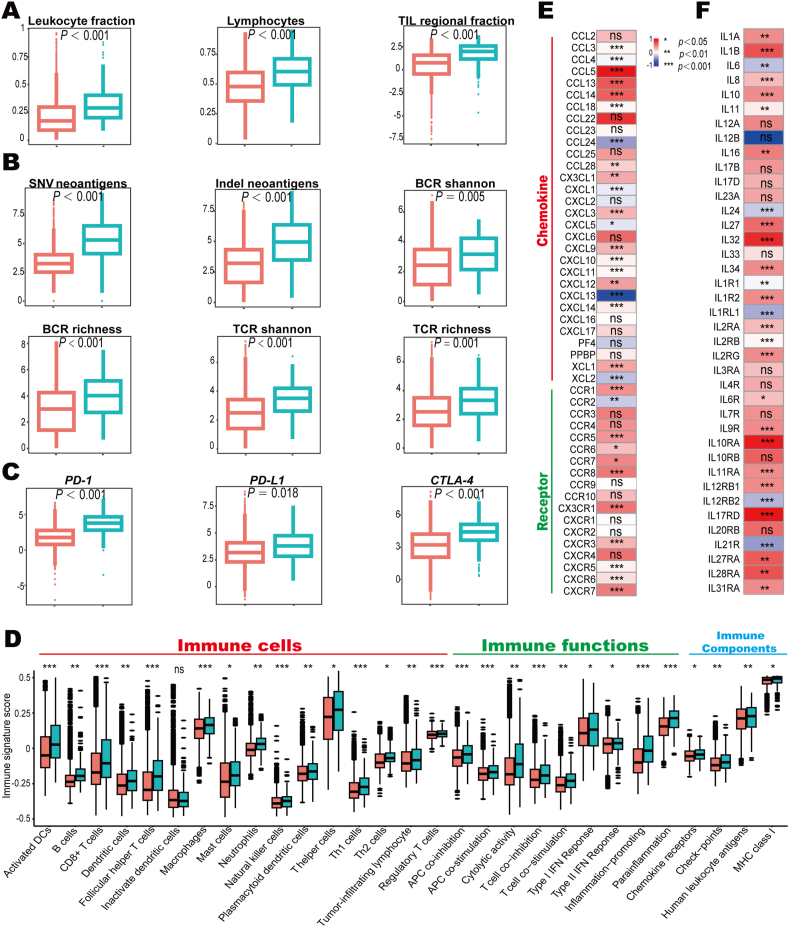
Comparison of extrinsic immunity in *ALK*-mutant and *ALK*-non-mutant tumors. **(A)** The immune cell infiltration was revealed by leukocyte fraction, lymphocyte fraction, and tumor-infiltrating lymphocyte fraction in *ALK-*mutant and *ALK*-non-mutant tumors. **(B)** The abundances of neoantigens and the diversity of TCR/BCR in *ALK-*mutant and *ALK*-non-mutant tumors. **(C)** mRNA expression levels of three major immune checkpoints, namely *PD-1*, *PD-L1*, and *CTLA-4*, in patients with *ALK-*mutant and *ALK-*non-mutant tumors. **(D)** Differences of 29 immune signatures estimated by ssGSEA between *ALK-*mutant and *ALK-*non-mutant tumors. **(E)** Comparison of 30 chemokines and 18 chemokine receptors between *ALK-*mutant and *ALK-*non-mutant tumors. **(F)** Expression differences of 39 immune-stimulators between *ALK-*mutant and *ALK-*non-mutant tumors. ALK, anaplastic lymphoma kinase; BCR, B cell receptor; SNV, single-nucleotide variant; TCR, T cell receptor; TIL, tumor-infiltrating lymphocyte; PD-L1, programmed cell death ligand 1; PD-1, programmed cell death protein 1; CTLA-4, cytotoxic T-lymphocyte-associated antigen 4.

The primary extrinsic immune features included the infiltration of immune cells, high diversity of BCRs/TCRs, activated immunogenicity of cancer cells contributing to the immune response, and high expression level of immune-stimulators and chemokines.^25^ *ALK* mutation was associated with higher levels of immune cell infiltration based on lymphocyte fraction, leukocyte fraction, and tumor-infiltrating lymphocyte fraction (Fig. 5A). Compared with *ALK*-non-mutant tumors, the abundances neoantigens (including single nucleotide variant neoantigen and indel neoantigen) and the diversity of TCR/BCR (estimated by TCR/BCR Shannon and TCR/BCR richness) were significantly up-regulated in *ALK*-mutant tumors (Fig. 5B). Additionally, the mRNA levels of key immune checkpoints (*CTLA-4*, *PD-1*, and *PD-L1*) were increased significantly in *ALK*-mutant tumors (Fig. 5C). The expression of 29 immune signatures, quantified by ssGSEA, revealed that the immune cell populations and immune activities were clearly enriched in *ALK*-mutant tumors (Fig. 5D). Moreover, *ALK* mutation was associated with higher levels of most chemokines and their receptors (Fig. 5E) and immune-stimulators (Fig. 5F).

## Discussion

It is well-established that the oncogenic drivers can impact the systemic and local immune landscape, hence participating in tumor evasion of immunosurveillance and subsequent immune response.^7^^,^^26^ However, data from subgroup analysis of one retrospective trial conducted in the US,^27^ one international multi-center retrospective trial (IMMUNOTARGET),^28^ and one phase 2 single-arm study (ATLANTIC)^29^ reveal that no patients with *ALK*-rearranged lung cancer respond to immune checkpoint inhibitors as single agents. Accordingly, the role of immunotherapy in patients with *ALK*-altered tumors is controversial currently. Considering there are significant heterogeneities in tumors harboring *ALK* rearrangement and *ALK* mutation,^5^^,^^6^^,^^8^ here we conduct a comprehensive bioinformatic and clinical analysis to systematically examine the association between *ALK* mutation and the efficacy of immune checkpoint blockade. Our data, for the first time to our knowledge, show that *ALK* mutation is associated with enhanced tumor immunity and favorable outcomes in pan-cancer immune checkpoint blockade.

The possible underlying mechanisms between *ALK* mutation and the benefit of immune checkpoint blockade are largely unclear. However, it should be pointed out that in normal physiological conditions, the expression of *ALK* is restricted to the nervous system, a highly immune-privileged organ.^5^ Hence, the ALK protein is a naturally potential antigen for the immune system. Similarly, tumor-specific *ALK* mutants may also be recognized as neoantigens in the body and trigger antibody responses in patients. Indeed, two immunogenic *ALK* epitopes (*p280-289* and *p375-386*) have been identified to elicit cytotoxic T cell responses in both *in vitro and in vivo* models.^10^ Specifically, they can induce an antigen-specific HLA-A2.1-restricted response, which kills endogenous ALK-expressing tumor cells effectively. Additionally, ALK release into the tumor microenvironment stimulates B-cells and tumor-associated macrophages, shaping a milieu conducive to immune evasion.^6^^,^^30^ More importantly, the occurrence of *ALK* mutation can result in the up-regulation of *PD-L1* expression.^6^^,^^30^ Through CRISPR/Cas9 library screening, Zhang et al elucidates that the induction of *PD-L1* is contingent upon the activation of the *ALK* oncoprotein, which subsequently activates signal transducer and activator of transcription 3 (*STAT3*), and a signalosome comprising growth factor receptor-bound protein 2 (*GRB2*)/Son of sevenless 1 (*SOS1*). This signalosome facilitates the activation of the *PI3K-AKT* and *MEK-ERK* signaling pathways. These signaling cascades culminate in the expression of *PD-L1* via the transcriptional activity of interferon regulatory factor 4 (IRF4) and basic leucine zipper ATF-like transcription factor 3 (BATF3) on the enhancer region of the *PD-L1* gene.^11^ Activated mechanistic target of rapamycin kinase (*mTOR*) facilitates the recruitment of *PD-L1* transcripts to active polysomes at the post-transcriptional level, thereby augmenting PD-L1 protein levels without a corresponding significant increase in mRNA levels.^31^ *STAT3* enhances *PD-L1* transcription by directly binding to the promoter region of cluster of differentiation 274 (*CD274*) gene.^32^ Recently, Nouri et al have discovered, through a kinome-wide screen of Hippo pathway regulators, that Yes-associated protein (*YAP*)/tafazzin (*TAZ*) play a crucial role in mediating *ALK*-induced up-regulation of *PD-L1*.^33^ Furthermore, *ALK* may contribute to enhanced immune evasion via the Janus kinase (*JAK*)*-STAT3-*large tumor suppressor kinase (*LATS*)*-YAP/TAZ-PD-L1* signaling pathway. Consistent with these results, our analysis conducted on the TCGA database also suggested that compared with *ALK*-non-mutant tumors, the expression of all major immune checkpoints, including *PD-L1*, was up-regulated in *ALK*-mutant tumors. Of note, the full implication of *ALK* mutation in immunotherapy remains elusive and needs in-depth examination.

Our study had several limitations. First, these eligible studies were conducted at various medical centers, and the tools used in analyzing sequencing data among these studies were different; the researchers had subjectivity in recording clinical outcomes. Our result is subject to any biases or errors derived from the original investigators. Second, *ALK*-mutant tumors were also identified in many other cancer types. However, due to the limited number of patients available, we cannot estimate the performance of *ALK* mutation in other tumors. Third, we conducted a retrospective study here; a head-to-head comparative perspective trial was warranted to validate these results. Fourth, due to the rarity of eligible patients, the anti-tumor activity of immunotherapy combinations in *ALK*-mutant tumors was not fully examined. It was well-established that chemotherapy, radiotherapy, or even immunotherapy itself could induce immunogenicity in patients.^3^^,^^34^ It is possible that more patients could benefit from immunotherapy when immune checkpoint inhibitors are combined with other agents. Fifth, the toxicity profile also played an important role in determining the optimal treatment strategy. However, it was difficult to carry out such an analysis to address this issue since reports of adverse events from the eligible cohorts were unavailable. Despite these limitations, with individual patient data derived from approximately 3000 subjects treated with immune checkpoint inhibitors, this is the first study to examine the performance of *ALK* mutation as a predictive biomarker in immunotherapy.

In summary, *ALK-*mutant tumors may be regarded as immunologically “hot” tumors as they were associated with both promoted intrinsic and extrinsic immunity. Moreover, *ALK* mutation is an independent biomarker for favorable outcomes in pan-cancer immune checkpoint blockade. These results have implications for treatment decision-making and developing immunotherapy for personalized care.

## CRediT authorship contribution statement

**Zhiyang Huang:** Writing – review & editing, Writing – original draft, Visualization, Validation, Software, Methodology, Investigation, Formal analysis, Data curation. **Jiajun Chen:** Writing – original draft, Visualization, Validation, Software, Project administration, Methodology, Investigation, Formal analysis, Data curation. **Yan Huang:** Writing – original draft, Visualization, Validation, Software, Resources, Methodology, Investigation, Formal analysis, Data curation. **Hong Zhao:** Writing – review & editing, Writing – original draft, Project administration, Funding acquisition. **Bin Zhao:** Writing – review & editing, Writing – original draft, Supervision, Conceptualization.

## Data availability

The datasets generated during and/or analyzed during the current study are available from the corresponding author upon reasonable request.

## Funding

This study was supported by the 10.13039/501100001809National Natural Science Foundation of China (No. 82373367). The sponsor does not participate in the collection, analysis, and interpretation of data; in the writing of the manuscript; and in the decision to submit the manuscript for publication.

## Conflict of interests

The authors declare no competing interests.
